# A Model to Incorporate the bHLH Transcription Factor OsIRO3 within the Rice Iron Homeostasis Regulatory Network

**DOI:** 10.3390/ijms23031635

**Published:** 2022-01-31

**Authors:** Oscar Carey-Fung, Martin O’Brien, Jesse T. Beasley, Alexander A. T. Johnson

**Affiliations:** 1 School of BioSciences, The University of Melbourne, Parkville, VIC 3010, Australia; ofung@student.unimelb.edu.au (O.C.-F.); jesse.beasley@unimelb.edu.au (J.T.B.); 2 Department of Animal, Plant and Soil Sciences, La Trobe University, Bundoora, VIC 3086, Australia; martin.obrien@latrobe.edu.au

**Keywords:** basic Helix–Loop–Helix, metal homeostasis, ferroptosis, gene editing

## Abstract

Iron (Fe) homeostasis in plants is governed by a complex network of regulatory elements and transcription factors (TFs), as both Fe toxicity and deficiency negatively impact plant growth and physiology. The Fe homeostasis network is well characterized in *Arabidopsis thaliana* and remains poorly understood in monocotyledon species such as rice (*Oryza sativa* L.). Recent investigation of the rice Fe homeostasis network revealed OsIRO3, a basic Helix–Loop–Helix (bHLH) TF as a putative negative regulator of genes involved in Fe uptake, transport, and storage. We employed CRISPR-Cas9 gene editing to target the *OsIRO3* coding sequence and generate two independent T-DNA-free, loss-of-function *iro3* mutants in rice cv. Nipponbare. The *iro3* mutant plants had similar phenotype under nutrient-sufficient conditions and had stunted growth under Fe-deficient conditions, relative to a T-DNA free, wild-type control (WT). Under Fe deficiency, *iro3* mutant shoots had reduced expression of Fe chelator biosynthetic genes (*OsNAS1, OsNAS2,* and *OsNAAT1*) and upregulated expression of an Fe transporter gene (*OsYSL15*), relative to WT shoots. We place our results in the context of the existing literature and generate a model describing the role of OsIRO3 in rice Fe homeostasis and reinforce the essential function of OsIRO3 in the rice Fe deficiency response.

## 1. Introduction

Plants respond to abiotic stresses by modulating the expression of target genes through the activity of transcription factors (TFs). The basic Helix–Loop–Helix (bHLH) TF family is one of the largest TF families in plants and plays important roles in anthocyanin biosynthesis, hormone signalling, and nutrient uptake [1,2,3]. All bHLH proteins contain a conserved DNA-binding basic region and two alpha helices separated by a variable loop region, and homo/heterodimerise with other TFs prior to binding DNA. Once dimerised, the bHLH protein complex can regulate transcription by binding to the E-box (5′-CANNTG-3′) of gene promoters, and the specificity of binding is determined by the E-box sequence and its affinity to residues within the basic and loop regions of bHLH domains [2,4,5,6]. In rice (*Oryza sativa* L.), up to 211 bHLH TFs have been annotated within 12 recognised subgroups (http://planttfdb.gao-lab.org/ accessed on 12 October 2020), including the subgroups Ib, IVb, and IVc bHLH TFs responsible for maintaining cellular iron (Fe) homeostasis and responding to changes in environmental Fe conditions [2]. One rice subgroup, IVb bHLH TF, iron-related transcription factor 3 (OsIRO3), is upregulated in response to environmental Fe deficiency, and overexpression of *OsIRO3* reduces the expression of Fe chelator biosynthetic genes and decreases plant growth and shoot Fe concentrations under Fe deficiency [7,8]. For these reasons, *OsIRO3* was initially characterised as a putative negative regulator of the Fe deficiency response in rice. Within the rice Fe homeostasis regulatory network, *OsIRO3* sits downstream of the master regulators iron deficiency-responsive element-binding factors 1 (OsIDEF1) and Hemerythrin motif-containing Really Interesting New Gene (RING) and zinc finger proteins 1 and 2 (OsHRZ1 and OsHRZ2), which are capable of sensing environmental Fe conditions [9,10,11]. The OsHRZ proteins are thought to regulate *OsIRO3* expression via 26S proteasome-mediated degradation of Positive Regulator of Iron homeostasis proteins (OsPRI1/OsbHLH60, OsPRI2/OsbHLH58 and OsPRI3/OsbHLH59), which are subgroup IVc bHLH TFs and function as upstream positive regulators of *OsIRO3* [12,13,14]. Putative targets for OsIRO3-mediated transcriptional repression include genes involved in Fe uptake and transport, *Yellow Stripe-Like 15* (*OsYSL15*), *Natural Resistance-Associated Macrophage Protein* (*OsNRAMP*), and *Iron Regulated Transporter* (*OsIRT1*), and biosynthetic genes of the Fe chelators nicotianamine (NA) and 2′-deoxymugineic acid (DMA), *Nicotianamine Synthase 1*, *2*, and *3* (*OsNAS1, OsNAS2,* and *OsNAS3*) and *Nicotianamine aminotransferase* 1 (*OsNAAT1*) [8,15].

Understanding the molecular function of *OsIRO3* was initially guided by characterisation of *POPEYE* (*AtPYE*), the ortholog of *OsIRO3* in *Arabidopsis thaliana* (Arabidopsis hereafter) [16]. Expression of *AtPYE* is positively regulated by subgroup IVc TFs and the AtPYE protein can dimerise with subgroup IVc TFs, suggesting that Fe regulatory bHLH TFs dynamically interact with each other [16,17,18]. Whether similar interactions occur between OsIRO3 and subgroup IVc (OsPRI) proteins in rice has yet to be determined. Under Fe deficiency, the AtPYE TF binds to the promoters of *Zinc Induced Facilitator 1* (*AtZIF1*), *Ferric Reductase Oxidase 3* (*AtFRO3*), and *AtNAS4* genes via a bHLH domain and represses their expression. The mode of AtPYE-mediated transcriptional repression is thought to occur via a conserved ethylene-responsive element binding factor-associated amphiphilic repression (EAR) motif within the AtPYE protein, although the exact function of this motif has yet to be confirmed [19]. Loss-of-function *AtPYE* mutants displayed reduced tolerance to Fe deficiency, Fe toxicity in leaves, and increased expression of *AtZIF1*, *AtFRO3* and *AtNAS4* genes relative to WT plants, providing the first evidence that AtPYE proteins are critical for regulating plant metal homeostasis [20,21,22].

Recent gene editing studies have further characterised the function of *OsIRO3* in rice using CRISPR-Cas9 technology to generate *OsIRO3* gene knockouts [23,24,25]. From these studies, the OsIRO3 protein was observed to both homodimerise and heterodimerise with OsbHLH062, a subgroup IVb bHLH TF involved in responding to cold stress in rice [24,26]. The OsIRO3 homodimer was observed to bind the promoter and repress expression of the *OsNAS3* gene, although the mode of OsIRO3-mediated transcriptional repression and the function of the OsIRO3-OsbHLH062 heterodimer was not determined [25]. Loss-of-function *OsIRO3* mutants displayed a similar phenotype to *AtPYE* mutants in Arabidopsis, with reduced tolerance to Fe deficiency, leaf Fe toxicity and necrosis, and transcriptional modifications. Together, these results confirm that IRO3 and PYE proteins share similar functions in monocots and eudicots, respectively, although the exact function of OsIRO3 in the rice Fe deficiency response remains unclear.

In this study, we contribute to the rapidly growing body of knowledge on *OsIRO3* function by generating novel CRISPR-Cas9-induced loss-of-function mutants of *OsIRO3* in cultivar Nipponbare. By incorporating our results with those from other related studies, we propose a model describing the holistic role of *OsIRO3* in regulating rice Fe homeostasis under both nutrient-sufficient and Fe-deficient conditions.

## 2. Results

### 2.1. Two Transgene-Free OsIRO3-Knockout Mutants Were Generated Using CRISPR-Cas9 Gene Editing

**Figure 1:**
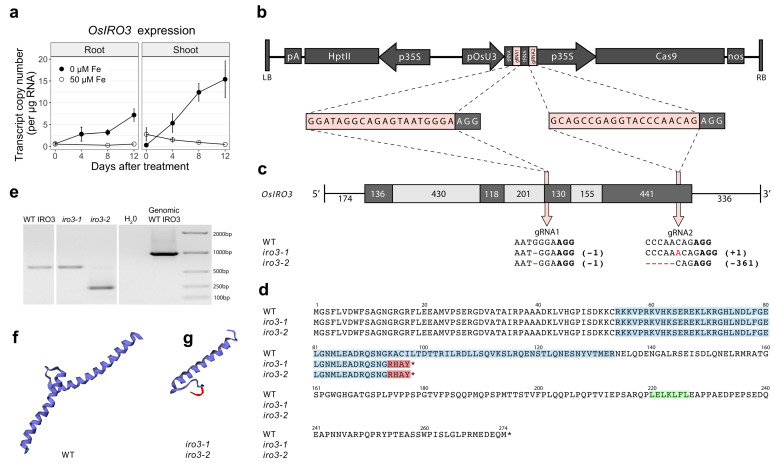
Endogenous *OsIRO3* expression and generation of loss of function *OsIRO3* mutations in rice cv. Nipponbare: (**a**) quantitative reverse transcription-PCR (qRT-PCR) analysis of endogenous OsIRO3 gene expression under Fe-deficient (0 µM Fe, black line) and Fe-sufficient (50 µM Fe, white line) conditions in root and shoot tissues. Error bars indicate the standard error of the mean of three biological replicates (*n* = 3), each with three technical replicates of qRT-PCR. (**b**) Schematic representation of the T-DNA construct: LB, left border; pA, CaMV poly(A) signal; HptII, hygromycin phosphotranferase II; p35S, CaMV 35S; pOsU3, rice U3 promoter; tRNA, transfer RNA; gRNA, guide RNA; Cas9, human codon optimized Streptococcus pyogenes Cas9; nos, nopaline synthase terminator; RB, right border. The gRNA sequences (pink) and PAM motif (black) are enlarged. (**c**) Gene structure of *OsIRO3* and target positions of gRNAs. The coding sequences (black boxes), introns (grey boxes), and untranslated regions (lines) of *OsIRO3* are depicted with length (base pairs) provided. The mutations at gRNA1 and gRNA2 in exon 3 and exon 4 of *OsIRO3*, respectively, are provided for both *iro3-1* and *iro3-2* mutants. (**d**) Predicted amino acid sequences of wild-type (WT), *iro3-1* and *iro3-2* OsIRO3 proteins. Residues within the conserved bHLH domain (blue), predicted EAR motif (green) and novel residues resulting from mutations at gRNA 1 (red) are highlighted. Stop codons are indicated with an asterisk. (**e**) Reverse transcription-PCR analysis of *OsIRO3* in 10 ng µL^−1^ of cDNA from WT, *iro3-1* and *iro3-2* young leaf tissues. Predicted protein structure of the OsIRO3 bHLH domain within (**f**) WT and (**g**) *iro3* mutants. The red ribbon indicates novel residues resulting from mutations at gRNA 1.

A CRISPR-Cas9 construct containing two guide RNA (gRNA) sequences targeting exon 3 (gRNA1) and exon 4 (gRNA2) of *OsIRO3* was transformed into rice to generate loss-of-function mutants of *OsIRO3* (Figure 1b,c and Figure S1). Two independent, homozygous, transgene-free *OsIRO3*-knockout mutants (hereafter referred to as *iro3-1* and *iro3-2*) were isolated. Sequencing revealed a single nucleotide deletion at gRNA1 in both the *iro3-1* and *iro3-2* mutants, causing a frameshift mutation and four novel residues in the peptide sequence prior to a premature stop codon (Figure 1c,d). At gRNA2, the *iro3-1* sequence contained a single nucleotide insertion, and the *iro3-2* sequence contained a 361-nucleotide deletion (Figure 1c). The mRNA transcripts of both *iro3-1* (typical length, fainter band) and *iro3-2* (smaller length, brighter band) were detected via reverse-transcription polymerase chain reaction (PCR) (Figure 1e). Both *iro3-1* and *iro3-2* mutant proteins were predicted to contain a truncated bHLH domain and lack a C-terminus relative to the WT IRO3 protein (Figure 1d,f,g). We also isolated a transgene-free wild-type (WT) line from a segregating population containing no mutation at either gRNA, which served as our control genotype in the experiments (hereafter referred to as iro3-wt).

### 2.2. The iro3 Mutants Had Normal Growth under Conditions of Nutrient Sufficiency and Alkalinity Stress, and Showed Hypersensitivity to Fe Deficiency Relative to WT

**Figure 2:**
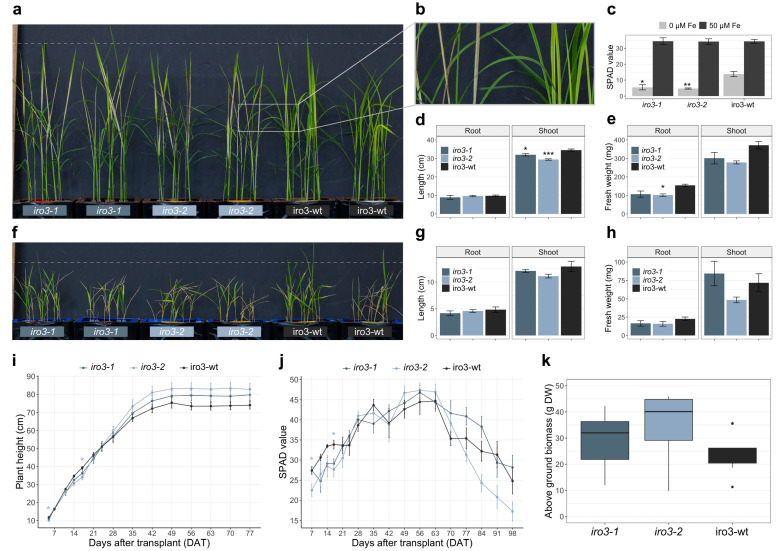
Phenotypic assessment of *iro3-1*, *iro3-2* and iro3-wt plants: (**a**) representative *iro3-1* (navy blue), *iro3-2* (light blue) and iro3-wt (black) plants at day 10 of the Fe deficiency treatment. A dashed line is presented at a height of 40 cm. (**b**) Necrotic leaves of *iro3-2* (left) and chlorotic leaves of iro3-wt (right). (**c**) SPAD of *iro3-1*, *iro3-2* and iro3-wt youngest leaves at day 11 of Fe deficiency (0 µM Fe, light grey) or Fe sufficiency (50 µM Fe, dark grey) treatment. (**d**,**e**) Length (cm) and fresh weight (mg) of *iro3-1*, *iro3-2* and iro3-wt roots and shoots under hydroponic Fe-deficient conditions. (**f**) Representative *iro3-1*, *iro3-2* and iro3-wt plants following 7 days of alkalinity stress and a 5-day recovery period. A dashed line is presented at a height of 20 cm. (**g**,**h**) Length (cm) and fresh weight (mg) of *iro3-1*, *iro3-2* and iro3-wt roots and shoots under hydroponic alkalinity stress conditions. Error bars indicate SEM of three biological replicates (*n* = 3) where each biological replicate is comprised of three representative plants. (**i**,**j**) Plant height (cm) and SPAD during the life cycle of *iro3-1*, *iro3-2* and iro3-wt plants grown under nutrient-sufficient soil conditions. (**k**) Above ground biomass (g dry weight) of *iro3-1*, *iro3-2* and iro3-wt plants at harvest under soil nutrient-sufficient conditions. Error bars indicate SEM of nine biological replicates (*n* = 9). Asterisks indicate significant differences between *iro3-1* and *iro3-2* with iro3-wt as determined by a one-way ANOVA (post hoc Tukey’s HSD test; * = *p* value ≤ 0.05; ** = *p* value ≤ 0.01; *** = *p* value ≤ 0.001).

Both *iro3-1* and *iro3-2* showed no phenotypic differences (tissue length and tissue weight) relative to iro3-wt under hydroponic nutrient-sufficient conditions (Figure S2). By day 10 of a hydroponic Fe deficiency treatment, *iro3-1* and *iro3-2* plants demonstrated necrosis and brown lesions on developing leaf tissues whilst iro3-wt plants demonstrated leaf chlorosis (Figure 2a,b). Following the Fe deficiency treatment, leaf SPAD was 2.50-fold (*p* = 0.012) and 2.91-fold (*p* = 0.008) lower in *iro3-1* and *iro3-2* plants, respectively, relative to iro3-wt (Figure 2c). Shoot length (cm) was 1.08-fold lower (*p* = 0.039) in *iro3-1* and 1.17-fold lower (*p* = 0.001) in *iro3-2* relative to iro3-wt in response to Fe deficiency (Figure 2d). Root fresh weight (g) was 1.44-fold lower (*p* = 0.052) in *iro3-1* and 1.52-fold lower (*p* = 0.035) in *iro3-2*, and shoot fresh weight (g) was 1.23-fold lower (*p* = 0.141) in *iro3-1* and 1.33-fold lower (*p* = 0.055) in *iro3-2* relative to iro3-wt in response to Fe deficiency (Figure 2e). Under conditions of hydroponic alkalinity stress (14 mM Na_2_CO_3_), both *iro3-1* and *iro3-2* showed no phenotypic differences (tissue length and tissue weight) relative to iro3-wt (Figure 2f,g,h and Table S1). Under soil nutrient-sufficient conditions, both *iro3-1* and *iro3-2* demonstrated no difference in plant height (cm) and SPAD relative to iro3-wt, with the exception of lower plant height at day 5 (*p* = 0.042) and day 17 (*p* = 0.040), and lower SPAD at day 7 (*p* = 0.017) and day 17 (*p* = 0.012) for *iro3-2* relative to iro3-wt (Figure 2i,j). Total above ground biomass (g DW) at harvest was 1.24-fold higher (*p* = 0.549) in *iro3-1* and 1.46-fold higher (*p* = 0.106) in *iro3-2* relative to iro3-wt. 

### 2.3. Expression of Fe Homeostasis Genes Was Upregulated in iro3 Mutant and WT Shoot Tissues in Response to Fe Deficiency and Suggests a Key Role for IRO3 in the Regulation of Rice Fe Homeostasis

**Figure 3:**
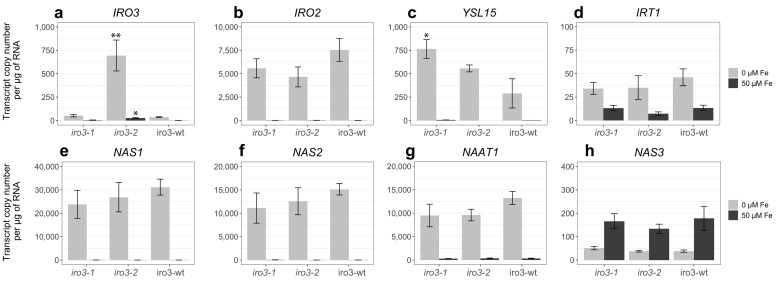
Expression of Fe homeostasis genes in *iro3-1*, *iro3-2* and iro3-wt shoot tissues under hydroponic conditions. The expression of (**a**) *OsIRO3*, (**b**) *OsIRO2*, (**c**) *OsYSL15*, (**d**) *OsIRT1*, (**e**) *OsNAS1*, (**f**) *OsNAS2*, (**g**) *OsNAAT1*, (**h**) *OsNAS3* genes in *iro3-1*, *iro3-2* and iro3-wt shoots following 11 days of Fe-deficient (0 µM Fe, light grey) or Fe-sufficient (50 µM Fe, dark grey) conditions. Error bars indicate SEM of three biological replicates (*n* = 3), where each biological replicate is comprised of three representative plants. Asterisks indicate significant differences between *iro3-1* and *iro3-2* with iro3-wt, as determined by a one-way ANOVA (post hoc Tukey’s HSD test; * = *p* value ≤ 0.05; ** = *p* value ≤ 0.01).

**Figure 4:**
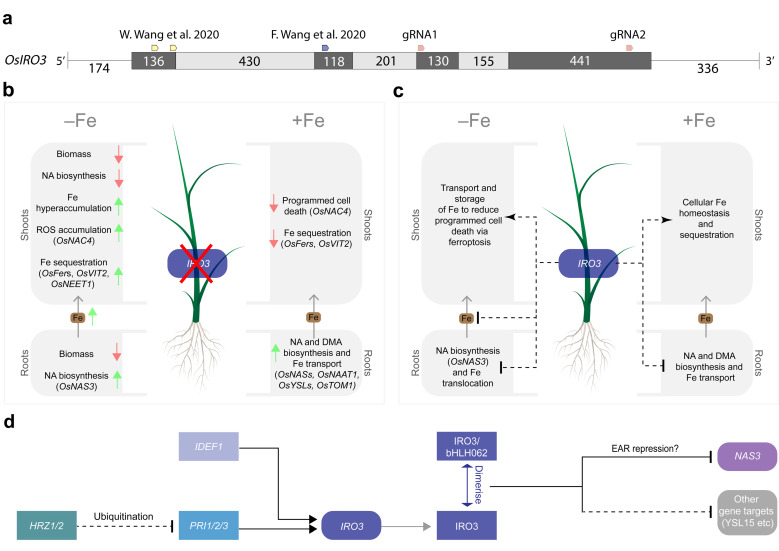
A model describing the role of *OsIRO3* within the rice Fe homeostasis regulatory network. (**a**) the location of five gRNA target sites used by three independent studies to generate loss-of-function *OsIRO3* rice mutants. (**b**) The effect of loss-of-function of *OsIRO3* on increasing (green arrows) or decreasing (red arrows) physiological processes in rice shoots and roots under Fe-deficient (−Fe) and Fe-sufficient (+Fe) conditions. (**c**) The multi-functional role of *OsIRO3* in rice shoots and roots under Fe-deficient (−Fe) and Fe-sufficient (+Fe) conditions. The process of translocating Fe (brown) from root to shoot tissues is indicated. (**d**) The proteins (rectangles) and genes (capsules) within the rice Fe homeostasis regulatory network, where the Fe homeostasis regulators (HRZ1/2, PRI1/2/3, IDEF1, IRO3, bHLH062 regulate the expression of downstream Fe homeostasis genes (NAS3, YSL15 and other gene targets). Direct (solid black line) and indirect (dashed black line) regulatory associations between transcription factors that either activate (arrow) or repress (blunt arrow) their targets are provided. The grey lines represent translation, and the blue line represents dimerization [24,25].

Transcriptional analysis of rice Fe homeostasis genes revealed upregulated expression of *OsIRO3, OsIRO2*, *OsYSL15, OsIRT1*, *OsNAS1*, *OsNAS2* and *OsNAAT1* in *iro3-1, iro3-2*, and iro3-wt shoot tissues by day 11 of a hydroponic Fe deficiency treatment (Figure 3a–g). Expression of *OsNAS3* was downregulated in *iro3-1, iro3-2*, and iro3-wt shoot tissues in response to Fe deficiency (Figure 3h). Expression of *OsIRO3* was similar between *iro3-1* and iro3-wt, and 20.0-fold (*p* = 0.013) and 21.4-fold (*p* = 0.007) higher in *iro3-2* relative to iro3-wt under Fe sufficiency and Fe deficiency, respectively (Figure 3a). Expression of *OsYSL15* was 2.64-fold higher (*p* = 0.049) in *iro3-1* and 1.92-fold higher (*p* = 0.270) in *iro3-2* relative to iro3-wt under Fe deficiency. These results were integrated into a model for *OsIRO3* function in regulating rice Fe homeostasis, as shown in Figure 4.

## 3. Discussion

Plants sense and respond to environmental Fe conditions through a signalling cascade of TFs in order to maximise Fe uptake and avoid Fe toxicity in tissues [27]. Functionally characterising regulatory components within this signalling cascade enhances our understanding of plant Fe stress tolerance and may lead to improvements in plant growth under Fe-limiting (e.g., alkaline) soil conditions (which represents up to 30% of global soils). The *OsIRO3* gene was initially characterised as a putative transcriptional repressor within the rice Fe homeostasis network, as overexpression of *OsIRO3* led to reduced tissue Fe concentrations and expression of Fe uptake and translocation genes [8]. More recently, the OsIRO3 TF was shown to negatively regulate *OsNAS3* gene expression by binding to the *OsNAS3* promoter via a conserved bHLH domain, although the exact mechanism behind OsIRO3-mediated transcriptional repression has yet to be determined [25]. A common form of transcriptional repression in plants occurs via EAR motifs which have consensus sequences LxLxL or DLNxxP. Functional characterisation of the subgroups IVb bHLH TF, AtbHLH11 demonstrated that the LxLxL EAR motif is essential for contacting the corepressor TOPLESS and recruiting a histone deacetylase to repress transcription repression [28,29]. We identified an LxLxL EAR motif near the C-terminus of the OsIRO3 protein (Figure 1d), and functional characterisation is now required to confirm that the EAR motif is essential for OsIRO3 proteins to negatively regulate target genes.

Including this study, three research groups have now generated unique loss-of-function *OsIRO3* mutants by disrupting the conserved bHLH domain (encoded within exons 2, 3 and 4) and EAR motif (encoded within exon 4) within the IRO3 protein (Figure 4a). Our two gRNA sequences targeted exons 3 and 4 of the *OsIRO3* gene and previous studies used gRNA sequences that targeted exons 1 and 2 (Figure 1b and Figure 4a) [24,25]. We successfully generated mutations at both gRNA sequence sites, and at gRNA2, these included both a single nucleotide insertion (*iro3-1*) and a 361-nucleotide deletion (*iro3-2*), although the effects of these mutations on OsIRO3 protein function are likely to be superseded by nonsense mutations at gRNA1 (Figure 1c,d). Expression of *OsIRO3* was detected in shoot tissues of *iro3-1* and *iro3-2* mutants, suggesting that *OsIRO3* transcripts are not degraded by nonsense-mediated RNA decay, which is a process that can occur in CRISPR-Cas9 gene-edited transcripts [30]. The significantly upregulated expression of *OsIRO3* in *iro3-2* mutant tissues relative to *iro3-1* and iro3-wt was consistent with initial RT-PCR analyses of *OsIRO3* expression in *iro3-1* (lighter band) and *iro3-2* (darker band) mutants and is likely due to mutational differences at gRNA2 (Figure 1a). Whether the large (361-nucleotide) deletion at the 3′ end of the *OsIRO3* transcript in *iro3-2* is responsible for these changes to *OsIRO3* transcription and/or transcript stability due to removal of a regulatory element is unknown. MicroRNA binding sites were recently observed in several Fe homeostasis rice genes, and whether *OsIRO3* contains similar elements will be the subject of future research. Despite these mutational differences, all *iro3* mutants demonstrated strikingly similar phenotypes under nutrient-sufficient and Fe-deficient conditions across the three studies, suggesting the OsIRO3 protein was rendered non-functional by each unique mutation. Under nutrient sufficient hydroponic and soil conditions, the *iro3-1* and *iro3-2* mutants demonstrated no phenotypical differences to iro3-wt, with the exception of slightly reduced shoot height and SPAD in *iro3-2* mutants during early stages of soil growth (Figure 2i–k and Figure S2). These slight reductions may indicate a temporary negative effect of *OsIRO3* mutations on early plant growth and future research is required to comprehensively evaluate *iro3* mutant agro-morphology under nutrient-sufficient soil conditions. Under Fe-deficient hydroponic conditions, the *iro3-1* and *iro3-2* mutants had reduced tissue length and biomass, and necrotic lesions on young leaves when compared to iro3-wt, suggesting that *iro3* mutant shoot tissues undergo programmed cell death (PCD) as a result of oxidative stress (Figure 2a–e). A similar phenotype was observed in an Arabidopsis *AtPYE* T-DNA insertion line, which demonstrated poor tolerance to Fe deficiency and elevated tissue Fe concentrations relative to WT plants [16]. Under conditions of excess Fe, reactive oxygen species (ROS) accumulate due to the Fenton reaction and can lead to Fe-toxicity-mediated PCD (also known as ferroptosis) [31,32]. Previous hydroponic Fe deficiency experiments demonstrated significantly higher shoot Fe concentration, shoot ROS concentrations, and activity of OsNAC4 (a TF that regulates PCD) in *iro3* mutants relative to WT plants [24,25,33]. In our study, upregulated expression of the NA-chelated Fe transporter *OsYSL15* was detected in *iro3* mutant shoots relative to iro3-wt shoots under hydroponic Fe deficiency (Figure 3c), suggesting that *iro3* mutants translocate excess Fe to shoot tissues under Fe deficiency, which eventually results in Fe toxicity and ferroptosis [34].

Based on the results of these three studies, we have developed a model to describe the role of *OsIRO3* in maintaining rice Fe homeostasis (Figure 4b–d). Under Fe sufficiency, OsIRO3 negatively regulates the expression of NA and DMA biosynthesis genes (*OsNAS1*, *OsNAS2*, *OsNAS3* and *OsNAAT1*) and Fe transporters (*OsYSL2*, *OsYSL15* and *OsTOM1*) in the roots, and promotes the expression of Fe sequestration genes (*OsFer1, OsFer2* and *OsVIT2*) in shoot tissues (Figure 4b,d) [8,25]. A small (but significant) increase in NA concentration and Fe uptake gene expression within *iro3* mutant root tissues under Fe sufficiency, coupled with no difference in *iro3* mutant shoot or root Fe concentrations, suggests that *IRO3* plays a minimal role in regulating Fe homeostasis in the absence of Fe stress (Figure 4c) [25]. By contrast, *OsIRO3* plays a critical role in limiting the translocation of Fe from root tissues and regulating excess Fe within shoot tissues under Fe deficiency (Figure 2a–c, Figure 3c and Figure 4b) [8,24,25]. The significant increase in NA concentration in *iro3* mutant root tissues under Fe deficiency is likely due to increased *OsNAS3* expression in *iro3* mutants relative to WT plants (Figure 4c) [25]. Among the three *OsNAS* genes of rice, *OsNAS3* demonstrates a unique expression pattern restricted to vascular cells of rice root and leaf tissues, suggesting that NA synthesised by OsNAS3 enzymes may be more responsible for transporting Fe throughout plant tissues compared to NA synthesised by OsNAS1 and OsNAS2 enzymes [35,36]. The high expression of OsNAS3 (and associated increase in NA concentration) in *iro3* mutant root tissues provides further evidence that *OsNAS3* is a direct target of *OsIRO3*-mediated transcriptional repression [25]. We hypothesize that under Fe deficiency, high concentrations of *OsNAS3*-synthesised NA in *iro3* mutants leads to excess root-to-shoot translocation of NA-chelated Fe, resulting in leaf Fe toxicity, ROS accumulation and ultimately ferroptosis. In response to shoot Fe toxicity, the *iro3* mutants upregulate the expression of Fe-sequestering genes (*OsFer1, OsFer2*, *OsNEET* and *OsVIT2*) and downregulate the expression of NA biosynthesis genes (*OsNAS1* and *OsNAS2*), which may account for the reduction in shoot NA concentrations observed in *iro3* mutants relative to WT plants (Figure 3e–f and Figure 4b) [24,25].

Soil Fe deficiency manifests under alkaline (pH > 8.0) soils, although we observed no differences in *iro3* mutant growth under hydroponic alkalinity stress conditions (Figure 2f–g and Table S1) [37]. The *OsIRO3* gene is typically upregulated under alkalinity stress due to plants sensing low environmental Fe, and rice varieties that contain a deletion in the 5′UTR of *OsIRO3* can tolerate alkalinity stress and exhibit low *OsIRO3* expression under alkalinity stress conditions [38]. Together, these results suggest that a knockdown (rather than a knockout) of *OsIRO3* may be more effective at improving rice abiotic stress tolerance. Alternatively, knocking out an essential *IRO3* gene within a species that has inbuilt genome redundancy (such as in hexaploid bread wheat) may mimic the effect of an *OsIRO3* knockdown, and represents a novel field of research for improving monocot abiotic stress tolerance [39].

## 4. Materials and Methods

### 4.1. Vector Construction and Plant Transformation

A CRISPR-Cas9 vector containing two guide RNA (gRNA) sequences, 5′-GGATAGGCAGAGTAATGGGA-3′ (gRNA 1) and 5’-GCAGCCGAGGTACCACAG-3′ (gRNA 2), which target exons 3 and 4 of the *OsIRO3* gene, respectively, was generated using the protocol previously described in [40]. Briefly, a human codon optimised SpCas9 gene under the transcriptional control of the cauliflower mosaic virus (CaMV) 35S promoter was inserted into the pCAMBIA1300 vector, using a Gibson cloning strategy (New England Biolabs, Ipswich, MA, USA). To allow for multiplex editing capability, the two gRNAs were assembled using a tRNA-gRNA system under transcriptional control of a U3 small nucleolar RNA promoter from rice and inserted between the SpCas9 cassette and a hygromycin phosphotransferase plant-selectable marker gene [41]. The final vector was mobilised into *Agrobacterium tumefaciens* (strain AGL1) by electroporation prior to rice transformation.

Rice transformation was performed as described in [42]. Briefly, embryonic rice calli (cv. Nipponbare) were cocultured with *Agrobacterium tumefaciens* and placed on hygromycin-containing media (50 mg/L) for selection of successful transformation events. Recovered rice transformants (T_0_) were rooted on P media for 3–4 weeks (12 h photoperiod, 28 °C) prior to greenhouse acclimation and transfer to soil.

### 4.2. Isolation of Homozygous, Independent, Transgene-Free, iro3 Mutant and WT Plants and In Silico Detection of Potential Off-Target Sites

Forty-two T_0_ transformation events were screened using Sanger sequencing for mutations at gRNA 1 and gRNA 2 with primers 5′-AGCTGTTTTGATCACAAGGCATTGCG-3′ and 5′-TCTGATTGGTTGGAGCTGATCTACTCAGG-3′. Out of the 36 T_0_ transformation events that contained mutations at either gRNA 1 or gRNA 2, 10 advanced to the T_1_ generation. To identify transgene-free T_1_ plants, all transformants were screened with primers 5′-CTTGTATGGAGCAGCAGACGC-3′ and 5′-CTATTTCTTTGCCCTCGGACG-3′ specific to the hygromycin resistance gene *hptII*. Detection of insertions/deletions (indels) at gRNA 1 was performed using the derived Cleaved Amplified Polymorphic Sequences (dCAPS) method with primers 5′-ATACAACAACCTTCTGCAGAAGCGGATAGCCAGAG-3′ and 5′-GGAGGGTGCTGTTTTCTTGCCGGAGAGATTTTAC-3′, followed by digestion with the restriction enzyme BstXI. Detection of a single guanine deletion at gRNA 1 was performed using Sanger sequencing. Detection of single nucleotide insertions at gRNA 2 in T_1_ plants was performed using the dCAPS method with primers 5′-GAACAATGTGGCACGACCGCAGCCGAGGTAACC-3′ and 5′-TGTGCTATCATCTATCGTGCTACCACTACATTTGC-3′, followed by digestion with the restriction enzyme BslI. Two T_2_ independent, homozygous mutant, transgene-free lines (hereafter referred to as *iro3-1* and *iro3-2*) and one T_2_ independent, homozygous WT, transgene-free line (hereafter referred to as iro3-wt) were selected for further analysis.

Potential off-target sites of the *OsIRO3* Cas9-gRNA complexes were predicted using Cas-OFFinder (http://www.rgenome.net/cas-offinder/ accessed 20 June 2019) with parameters: mismatch number ≤ 2, DNA bulge size ≤ 0, and RNA bulge size ≤ 0, and no off-target sequences were identified.

### 4.3. Soil and Hydroponic Growth Conditions

Seeds of *iro3-1*, *iro3-2*, and iro3-wt were surface sterilised in ethanol (C_2_H_5_OH, 70%) and bleach (NaOCl, 30%) solutions separately for 30 min, rinsed with 18 MΩ H_2_O, and germinated on moist filter paper (12 h photoperiod, 28 °C day/24 °C night) for either four or seven days for hydroponic or soil experiments, respectively. 

Hydroponic experiments utilised nutrient solutions containing all essential macronutrients NH_4_NO_3_ (5 mM), KNO_3_ (5 mM), Ca(NO_3_)_2_∙4H_2_O (2 mM), MgSO_4_∙7H_2_O (2 mM), and KH_2_PO_4_ (0.1 mM), and micronutrients H_3_BO_3_ (50 µM), MnCl_2_∙4H_2_O (5 µM), ZnSO_4_∙7H_2_O (5 µM), CuSO_4_∙5H_2_O (0.5 µM), NaMoO_4_∙2H_2_O (0.1 µM) and NaFe^3+^EDTA (50 µM). For the hydroponic experiments, nine germinated rice seedlings were transferred from filter paper to a hydroponic box (630 mL) containing nutrient solution and grown under optimal conditions in a growth chamber (12 h photoperiod, 28 °C day/24 °C night, 70% humidity). The hydroponic solution was replaced every 2–3 days and pH was maintained at 5.5. Each hydroponic box represented a biological replicate, and three biological replicates were established for *iro3-1*, *iro3-2*, and iro3-wt per treatment. For the Fe deficiency experiment, rice plants were exposed to six days of nutrient-sufficient conditions prior to eleven days of either Fe-sufficient (50 µM NaFe^3+^EDTA) or Fe-deficient (0 µM NaFe^3+^EDTA) conditions. After eleven days of Fe sufficiency or Fe deficiency, three plants from each replicate were combined to measure SPAD (youngest leaves) and harvest shoot and root tissues for phenotypic assessment. For the alkalinity stress experiment, rice plants were exposed to four days of nutrient-sufficient conditions, seven days of either alkalinity stress (14 mM Na_2_CO_3_) or control (0 mM Na_2_CO_3_) conditions, and a five day recovery period of nutrient-sufficient (0 mM Na_2_CO_3_) conditions, as previously described [38]. Following the recovery period, shoot and root tissues were harvested separately, and alkalinity tolerance was assessed phenotypically based on criteria previously described [43].

For the soil experiments, rice plants were sown in black plastic pots containing 1 L of soil mixture (one part fine sand, propagating sand, and potting media, and two parts vermiculite) as well as Osmocote^®^ Exact Standard 8–9 M fertiliser (6 g/L). Plants were maintained under glasshouse conditions (26 °C and 70% humidity) at The University of Melbourne (Parkville, VIC 3010, Australia).

### 4.4. Protein Modelling

Amino acid sequences of the OsIRO3 protein bHLH domain from *iro3* mutants and iro3-wt were submitted to the Phyre2 web portal (http://www.sbg.bio.ic.ac.uk/phyre2 accessed on 2 September 2019) for protein modelling, prediction and analysis [44]. The protein models in Figure 1 were generated and annotated using EzMol molecular display wizard http://www.sbg.bio.ic.ac.uk/ezmol/ accessed on 2 September 2019) based on outputs from Phyre2 software v2.0 [45].

### 4.5. Quantitative Reverse Transcription-PCR (qRT-PCR) Analysis of Endogenous OsIRO3 Expression in WT Plants and Fe Homeostasis Genes in iro3 Mutants and iro3-wt Plants

Quantitative RT-PCR analysis of endogenous *OsIRO3* expression in rice (cv. Nipponbare) shoot and root tissues (as shown in Figure 1a) was performed as previously described [46]. Briefly, shoot and root tissues (excluding the crown) from three, 13-day old, wild-type rice seedlings were harvested at days 0, 4, 8, and 12 of exposure to either hydroponic Fe-sufficient (50 µM NaFe^3+^EDTA) or hydroponic Fe-deficient (0 µM NaFe^3+^EDTA) conditions and combined to represent one biological replicate. Total RNA was extracted from homogenized samples using TRIzol (Thermo Fisher, Carlsbad, CA, USA) and purified using the RNeasy^Ⓡ^ Plant Minikit (Qiagen, Germantown, MD, USA) according to manufacturer’s specifications. Genomic DNA was removed using a DNAse I treatment kit (Promega, Madison, WI, USA) and reverse transcription was performed using a cDNA synthesis kit (Bioline, London, UK). Each biological replicate (10 ng µL^−1^ of cDNA) was analysed in triplicate and transcripts were quantified against a standard curve of three replicates of 10-fold serial dilutions (10^2^–10^8^) of purified template for each primer pair. The geometric mean expression of housekeeping genes: actin (*OsACT1*), elongation factor 1-delta 2 (*OsELF1*) and genevestigator gene 2 (*OsP2*) for shoot tissues and *OsACT1, OsELF1* and elongation factor 1-alpha (*OsEF-1a*) for root tissues was used to normalize qRT-PCR expression data as previously described [47,48,49]. To measure the expression of Fe homeostasis genes in *iro3* mutants and iro3-wt plants, qRT-PCR analysis on shoot tissues from three plants of each *iro3-1*, *iro3-2*, and iro3-wt replicate after eleven days of Fe sufficiency or Fe deficiency was performed as outlined above. The primer sequences used to measure endogenous *OsIRO3* and Fe homeostasis gene expression are provided in Table S2.

### 4.6. Statistical Analysis

Statistically significant differences between *iro3-1* and *iro3-2* with iro3-wt were determined using a one-way ANOVA followed by the post hoc Tukey’s HSD test (5% level of significance) and calculated in Rstudio software (https://rstudio.com/ v3.6.3 accessed 11 August 2020). All graphs were generated using the ggplot2 software package in RStudio (https://ggplot2.tidyverse.org/ accessed 11 August 2020).

## Data Availability

Data generated or analysed during this study are included in this published article (and its Supplementary Information files), and raw data is openly available in FigShare at https://doi.org/10.26188/15059406.

## Supplementary Materials

The following are available online at https://www.mdpi.com/article/10.3390/ijms23031635/s1.

## References

[1] Ludwig S.R., Habera L.F., Dellaporta S.L., Wessler S.R. (1989). Lc, a member of the maize R gene family responsible for tissue-specific anthocyanin production, encodes a protein similar to transcriptional activators and contains the myc-homology region. Proc. Natl. Acad. Sci. USA.

[2] Heim M.A., Jakoby M., Werber M., Martin C., Weisshaar B., Bailey P.C. (2003). The Basic Helix-Loop-Helix Transcription Factor Family in Plants: A Genome-Wide Study of Protein Structure and Functional Diversity. Mol. Biol. Evol..

[3] Goossens J., Mertens J., Goossens A. (2017). Role and functioning of bHLH transcription factors in jasmonate signalling. J. Exp. Bot..

[4] Toledo-Ortiz G., Huq E., Quail P.H. (2003). The Arabidopsis Basic/Helix-Loop-Helix Transcription Factor Family. Plant Cell.

[5] Grove C.A., De Masi F., Barrasa M.I., Newburger D.E., Alkema M.J., Bulyk M.L., Walhout A.J.M. (2009). A Multiparameter Network Reveals Extensive Divergence between C. elegans bHLH Transcription Factors. Cell.

[6] Pires N., Dolan L. (2010). Origin and Diversification of Basic-Helix-Loop-Helix Proteins in Plants. Mol. Biol. Evol..

[7] Zheng L., Huang F., Narsai R., Wu J., Giraud E., He F., Cheng L., Wang F., Wu P., Whelan J. (2009). Physiological and Transcriptome Analysis of Iron and Phosphorus Interaction in Rice Seedlings. Plant Physiol..

[8] Zheng L., Ying Y., Wang L., Wang F., Whelan J., Shou H. (2010). Identification of a novel iron regulated basic helix-loop-helix protein involved in Fe homeostasis in Oryza sativa. BMC Plant Biol..

[9] Kobayashi T., Ogo Y., Itai R.N., Nakanishi H., Takahashi M., Mori S., Nishizawa N.K. (2007). The transcription factor IDEF1 regulates the response to and tolerance of iron deficiency in plants. Proc. Natl. Acad. Sci. USA.

[10] Ogo Y., Kobayashi T., Nakanishi R., ‡1 I., Nakanishi H., Kakei Y., Takahashi M., Toki S., Mori S., Nishizawa N.K. (2008). A Novel NAC Transcription Factor, IDEF2, That Recognizes the Iron Deficiency-responsive Element 2 Regulates the Genes Involved in Iron Homeostasis in Plants. J. Biol. Chem..

[11] Kobayashi T., Nagasaka S., Senoura T., Itai R.N., Nakanishi H., Nishizawa N.K. (2013). Iron-binding haemerythrin RING ubiquitin ligases regulate plant iron responses and accumulation. Nat. Commun..

[12] Zhang H., Li Y., Yao X., Liang G., Yu D. (2017). Positive regulator of iron homeostasis1, OsPRI1, Facilitates Iron Homeostasis 1. Plant Physiol..

[13] Zhang H., Li Y., Pu M., Xu P., Liang G., Yu D. (2020). Oryza sativa positive regulator of iron deficiency response 2 (OsPRI2) and OsPRI3 are involved in the maintenance of Fe homeostasis. Plant Cell Environ..

[14] Kobayashi T., Ozu A., Kobayashi S., An G., Jeon J.S., Nishizawa N.K. (2019). OsbHLH058 and OsbHLH059 transcription factors positively regulate iron deficiency responses in rice. Plant Mol. Biol..

[15] Kobayashi T., Nishizawa N.K. (2012). Iron Uptake, Translocation, and Regulation in Higher Plants. Annu. Rev. Plant Biol..

[16] Long T.A., Tsukagoshi H., Busch W., Lahner B., Salt D.E., Benfey P.N. (2010). The bHLH Transcription Factor POPEYE Regulates Response to Iron Deficiency in Arabidopsis Roots. Plant Cell.

[17] Zhang J., Liu B., Li M., Feng D., Jin H., Wang P., Liu J., Xiong F., Wang J., Wang H. (2015). Bin The bHLH transcription factor bHLH104 interacts with IAA-LEUCINE RESISTANT3 and modulates iron homeostasis in arabidopsis. Plant Cell.

[18] Liang G., Zhang H., Li X., Ai Q., Yu D. (2017). bHLH transcription factor bHLH115 regulates iron homeostasis in Arabidopsis thaliana. J. Exp. Bot..

[19] Tissot N., Robe K., Gao F., Grant-Grant S., Boucherez J., Bellegarde F., Maghiaoui A., Marcelin R., Izquierdo E., Benhamed M. (2019). Transcriptional integration of the responses to iron availability in Arabidopsis by the bHLH factor ILR3. New Phytol..

[20] Mukherjee I., Campbell N.H., Ash J.S., Connolly E.L. (2006). Expression profiling of the Arabidopsis ferric chelate reductase (FRO) gene family reveals differential regulation by iron and copper. Planta.

[21] Haydon M.J., Cobbett C.S. (2007). A novel major facilitator superfamily protein at the tonoplast influences zinc tolerance and accumulation in Arabidopsis. Plant Physiol..

[22] Klatte M., Schuler M., Wirtz M., Fink-Straube C., Hell R., Bauer P. (2009). The analysis of arabidopsis nicotianamine synthase mutants reveals functions for nicotianamine in seed iron loading and iron deficiency responses. Plant Physiol..

[23] Zhang Y., Massel K., Godwin I.D., Gao C. (2018). Applications and potential of genome editing in crop improvement. Genome Biol..

[24] Wang F., Itai R.N., Nozoye T., Kobayashi T., Nishizawa N.K., Nakanishi H. (2020). The bHLH protein OsIRO3 is critical for plant survival and iron (Fe) homeostasis in rice (Oryza sativa L.) under Fe-deficient conditions. Soil Sci. Plant Nutr..

[25] Wang W., Ye J., Ma Y., Wang T., Shou H., Zheng L. (2020). OsIRO3 Plays an Essential Role in Iron Deficiency Responses and Regulates Iron Homeostasis in Rice. Plants.

[26] Wang Y.J., Zhang Z.G., He X.J., Zhou H.L., Wen Y.X., Dai J.X., Zhang J.S., Chen S.Y. (2003). A rice transcription factor OsbHLH1 is involved in cold stress response. Theor. Appl. Genet..

[27] Gao F., Robe K., Gaymard F., Izquierdo E., Dubos C. (2019). The Transcriptional Control of Iron Homeostasis in Plants: A Tale of bHLH Transcription Factors?. Front. Plant Sci..

[28] Kagale S., Rozwadowski K. (2011). EAR motif-mediated transcriptional repression in plants: An underlying mechanism for epigenetic regulation of gene expression. Epigenetics.

[29] Li Y., Lei R., Pu M., Cai Y., Lu C., Li Z., Liang G. (2020). bHLH11 negatively regulates Fe homeostasis by its EAR motifs recruiting corepressors in Arabidopsis. bioRxiv.

[30] Popp M.W., Maquat L.E. (2016). Leveraging rules of nonsense-mediated mRNA decay for genome engineering and personalized medicine. Cell.

[31] Fenton H.J.H. (1894). Oxidation of tartaric acid in presence of iron. J. Chem. Soc. Trans..

[32] Conlon M., Dixon S.J. (2017). Ferroptosis-like death in plant cells. Mol. Cell. Oncol..

[33] Kaneda T., Taga Y., Takai R., Iwano M., Matsui H., Takayama S., Isogai A., Che F.S. (2009). The transcription factor OsNAC4 is a key positive regulator of plant hypersensitive cell death. EMBO J..

[34] Inoue H., Kobayashi T., Nozoye T., Takahashi M., Kakei Y., Suzuki K., Nakazono M., Nakanishi H., Mori S., Nishizawa N.K. (2009). Rice OsYSL15 is an iron-regulated iron (III)-deoxymugineic acid transporter expressed in the roots and is essential for iron uptake in early growth of the seedlings. J. Biol. Chem..

[35] Inoue H., Higuchi K., Takahashi M., Nakanishi H., Mori S., Nishizawa N.K. (2003). Three rice nicotianamine synthase genes, OsNAS1, OsNAS2, and OsNAS3 are expressed in cells involved in long-distance transport of iron and differentially regulated by iron. Plant J..

[36] Aung M.S., Masuda H., Nozoye T., Kobayashi T., Jeon J.-S., An G., Nishizawa N.K. (2019). Nicotianamine Synthesis by OsNAS3 Is Important for Mitigating Iron Excess Stress in Rice. Front. Plant Sci..

[37] de Caritat P.A., Cooper M.A., Wilford J.A. (2011). The pH of Australian soils: Field results from a national survey. Soil Res..

[38] Li N., Zheng H., Cui J., Wang J., Liu H., Sun J., Liu T., Zhao H., Lai Y., Zou D. (2019). Genome-wide association study and candidate gene analysis of alkalinity tolerance in japonica rice germplasm at the seedling stage. Rice.

[39] Carey-Fung O., Beasley J.T., Johnson A.A.T. (2021). Annotation and Molecular Characterisation of the TaIRO3 and TaHRZ Iron Homeostasis Genes in Bread Wheat (Triticum aestivum L.). Genes.

[40] Naim F., Dugdale B., Kleidon J., Brinin A., Shand K., Waterhouse P., Dale J. (2018). Gene editing the phytoene desaturase alleles of Cavendish banana using CRISPR/Cas9. Transgenic Res..

[41] Xie K., Minkenberg B., Yang Y. (2015). Boosting CRISPR/Cas9 multiplex editing capability with the endogenous tRNA-processing system. Proc. Natl. Acad. Sci. USA.

[42] Sallaud C., Meynard D., Van Boxtel J., Gay C., Bès M., Brizard J.P., Larmande P., Ortega D., Raynal M., Portefaix M. (2003). Highly efficient production and characterization of T-DNA plants for rice (Oryza sativa L.) functional genomics. Theor. Appl. Genet..

[43] Bado S., Forster B.P., Ghanim A.M.A., Jankowicz-Cieslak J., Berthold G., Luxiang L., Bado S., Forster B.P., Ghanim A.M.A., Jankowicz-Cieslak J. (2016). Protocol for Screening for Salt Tolerance in Rice. Protocols for Pre-Field Screening of Mutants for Salt Tolerance in Rice, Wheat and Barley.

[44] Kelley L.A., Mezulis S., Yates C.M., Wass M.N., Sternberg M.J.E. (2015). The Phyre2 web portal for protein modeling, prediction and analysis. Nat. Protoc..

[45] Reynolds C.R., Islam S.A., Sternberg M.J.E. (2018). EzMol: A Web Server Wizard for the Rapid Visualization and Image Production of Protein and Nucleic Acid Structures. J. Mol. Biol..

[46] Beasley J.T., Bonneau J.P., Sánchez-Palacios J.T., Moreno-Moyano L.T., Callahan D.L., Tako E., Glahn R.P., Lombi E., Johnson A.A.T. (2019). Metabolic engineering of bread wheat improves grain iron concentration and bioavailability. Plant Biotechnol. J..

[47] Vandesompele J., De Preter K., Pattyn F., Poppe B., Van Roy N., De Paepe A., Speleman F. (2002). Accurate normalization of real-time quantitative RT-PCR data by geometric averaging of multiple internal control genes. Genome Biol..

[48] Selby-Pham J., Lutz A., Moreno-Moyano L.T., Boughton B.A., Roessner U., Johnson A.A.T. (2017). Diurnal Changes in Transcript and Metabolite Levels during the Iron Deficiency Response of Rice. Rice.

[49] de Castro dos Santos F.I., Marini N., dos Santos R.S., Hoffman B.S.F., Alves-Ferreira M., de Oliveira A.C. (2018). Selection and testing of reference genes for accurate RT-qPCR in rice seedlings under iron toxicity. PLoS ONE.

