# Supplementary material for: A Model to Incorporate the bHLH Transcription Factor OsIRO3 within the Rice Iron Homeostasis Regulatory Network
Source: Int J Mol Sci. 2022 Jan 31;23(3):1635. doi: 10.3390/ijms23031635 (PMC8835859; doi:10.3390/ijms23031635)
Supplement: Supplementary file 1 [file ijms-23-01635-s001.zip › ijms-1555918-supplementary.pdf]

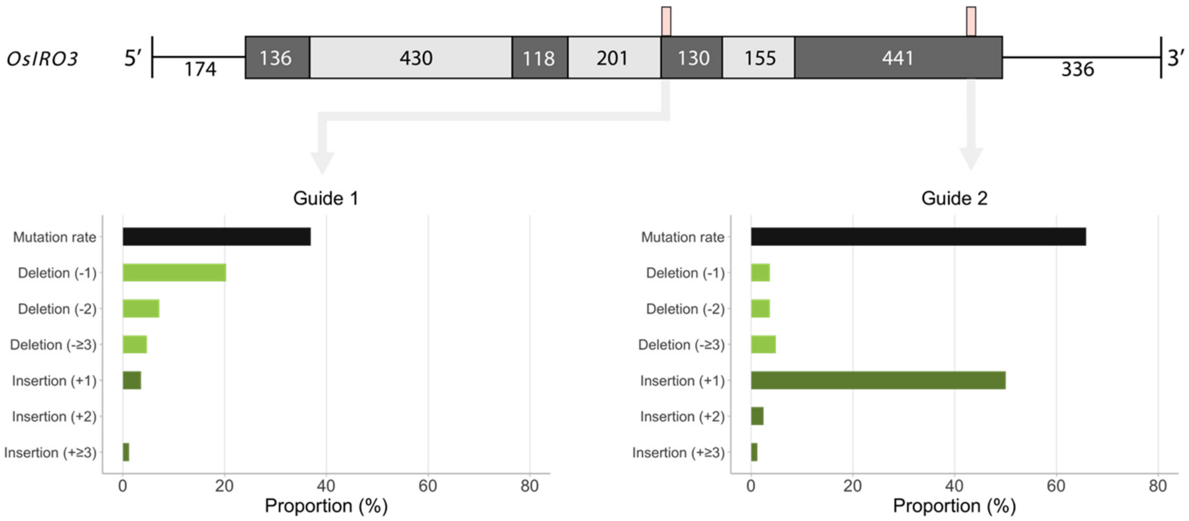

Figure S1. The proportion of different mutation rates at each guide RNA site of the OsIRO3 gene. The gene structure of OsIRO3 and target positions of guide RNAs (gRNA) are shown (pink). The coding sequences (black boxes), introns (grey boxes), and untranslated regions (lines) of OsIRO3 are depicted with length (base pairs) provided. The corresponding proportion of different mutations achieved at gRNA 1 and gRNA 2 of the OsIRO3 gene are calculated from 42 recovered T0 rice plants following transformation.

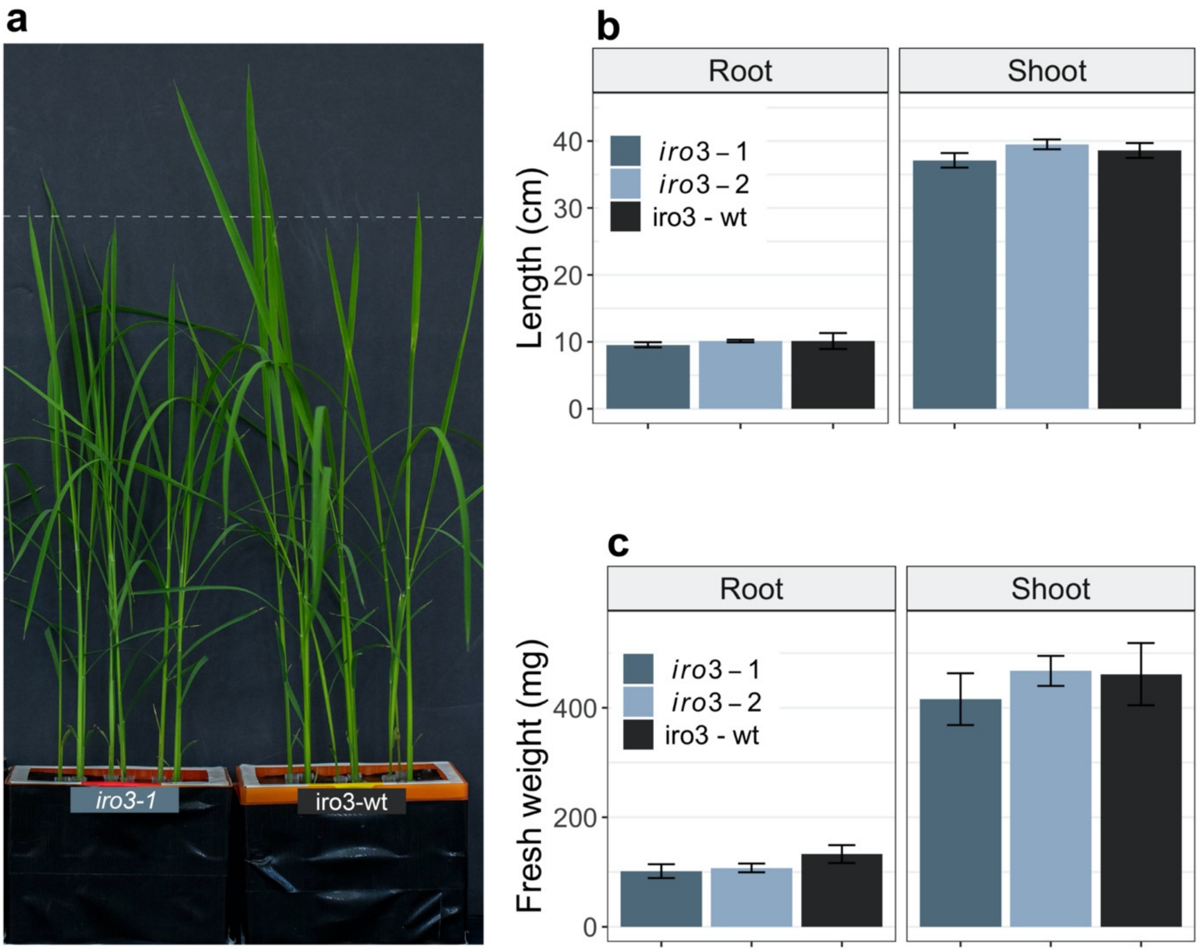

Figure S2. Phenotypic assessment of *iro3-1*, *iro3-2* and iro3-wt plants grown under hydroponic alkalinity stress conditions. (**a**) Representative iro3-1 (navy blue) and iro3-wt (black) plants following 7 days of alkalinity stress and a 5 day recovery period. A dashed line is presented at a height of 40 cm. (**b**,**c**) Length (cm) and fresh weight (mg) of iro3-1, iro3-2 and iro3-wt roots and shoots under hydroponic alkalinity stress conditions. Error bars indicate SEM of three biological replicates (n = 3) where each biological replicate is comprised of three representative plants.

Table S1. Indicators of alkalinity stress in *iro3-1*, *iro3-2* and iro3-wt plants.

Genotype	Alkalinity Tolerance Score	Proportion of dead leaves	Total no. of dead plants
iro3-1	7.00	0.47	10
iro3-2	7.67	0.63	11
iro3-wt	7.00	0.47	8

Table S2. Primers used for quantitative RT-PCR analysis.

Gene	Forward primer (5' – 3')	Reverse primer (3' – 5')	PCR product length (bp)	Annealing temperature (C°)
OsIRO3	TCCATGGGCCTATCTCTGAC	TGCCTATCCGCTTCTAGCAT	139	60
OsIRO2	TGGTTCAGGTCAGCTTGTTG	GTTCCTTCACTTCTCTGAAGATGG	166	60
OsYSL15	GGAACAAGATGAACAAGAAGGAG	CGAGAGCAAGGATAGAAGAAGG	108	59
OsIRT1	AGGTCGGTGCTCGTCTTCT	TGTCCCTGTACACCCTGGTC	85	60
OsNAS1	AACTCCGTCATCGTCGCC	GCAAACTCTTCTGCCTTCTCA	165	61
OsNAS2	GTCATCGTCGCTCGCAAG	GAACTCTTCCGCCTTCTGG	152	61
OsNAAT1	GTCCTCACAAGCCCGAAG	TTCATTCCCAGCACACTCC	148	59
OsNAS3	GGTGATCAACTCCGTCATCA	CTACGAGGAGGGCAGCTTCT	187	60
OsACT1	CTATGTTCCCTGGCATTGCT	ACATCTGCTGGAATGTGCTG	156	60
OsELF1	GAAAGCAGCAGAAGAACGGG	TCCTCAAGCTTTGCCATGTCT	126	60
OsP2	CTCCTTCTGGCCCTGTCTCT	CCCCCTAGAATGTTCCCTGA	121	61
OsEF-1a	CTGCTGCAACAAGATGGATG	CAGTCAAGGTTGGTGGACCT	180	60
**OsIRO3*	*GCGAGCTGGGTAATATGCTAGA	*GGGCTCAATGACTGTTGGTT	825	60

The asterisks indicate that these primers were used to detect the mRNA OsIRO3 transcript using reverse transcription-PCR analysis.
